# Afibercept treatment for macular edema with and without serous retinal detachment due to branch retinal vein occlusion

**DOI:** 10.5935/0004-2749.20230019

**Published:** 2023

**Authors:** Saadet Gültekin Irgat, Fatih Özcura

**Affiliations:** 1 Department of Ophthalmology, Kutahya Health Sciences University School of Medicine, Kutahya, Turkey

**Keywords:** Intravitreal injections, Aflibercept, Retinal vein occlusion, Macular edema, Retinal detachment, Injeções intravítreas, Aflibercepte, Oclusão da veia retiniana, Edema macular, Descolamento retiniano

## Abstract

**Purpose:**

To evaluate the effectiveness of intravitreal aflibercept treatment for macular edema with and without serous retinal detachment due to branch retinal vein occlusion.

**Methods:**

Thirty-seven eyes with branch retinal vein occlusion treated with intravitreal aflibercept injection for macular edema were evaluated retrospectively. The patients were divided into two groups according to whether they showed serous retinal detachment on spectral domain optical coherence tomography. Pro re nata regimen was applied after 1 dose of intravitreal aflibercept injection. After the initial injection, control treatments were administered at months 1, 2, 3, 6, and 12. The best-corrected visual acuity and central macular thickness were measured.

**Results:**

Fifteen patients had serous retinal detachment, and 22 with macular edema only (non-serous retinal detachment). The central macular thickness was significantly greater in the group with than in the group without serous retinal detachment (811.73 ± 220.68 µm and 667.90 ± 220.68 µm, respectively, p=0.04). The difference between the groups disappeared from the third month. The central macular thickness was similar between the two groups at the last control treatment (407.27 ± 99.08 µm and 376.66 ± 74.71 µm, p=0.66). The best-corrected visual acuity increased significantly in both groups. No significant difference was found between the two groups in terms of the best-corrected visual acuities at baseline and the final control.

**Conclusion:**

The intravitreal aflibercept treatment was highly effective in improving best-corrected visual acuity and central macular thickness in patients with branch retinal vein occlusion-induced macular edema independent of serous retinal detachment.

## Abstract (pt)

**Objetivo:**

Avaliar a eficácia do tratamento com aflibercepte intravítreo para edema macular devido à oclusão de um ramo da veia retiniana, com e sem descolamento seroso da retina.

**Métodos:**

Foram analisados retrospectivamente 37 olhos com oclusão de um ramo da veia retiniana, tratados com injeção intravítrea de aflibercepte para edema macular. Os pacientes foram divididos em dois grupos, de acordo com a presença ou ausência de um descolamento seroso de retina na tomografia de coerência óptica (SD-OCT). Um regime *pro re nata* foi seguido após 1 injeção intravítrea de aflibercepte. Após a injeção, foram realizadas consultas de acompanhamento nos meses 1, 2, 3, 6 e 12. Foram medidas a melhor acuidade visual corrigida e a espessura macular central.

**Resultados:**

Houve 15 pacientes com descolamento seroso de retina e 22 pacientes com apenas edema macular (descolamento não seroso de retina). A espessura macular central foi significativamente maior no grupo com descolamento seroso de retina do que no grupo com descolamento não seroso de retina (respectivamente, 811,73 ± 220,68 µm e 667,90 ± 220,68 µm; p=0,04). A diferença desapareceu a partir do terceiro mês. A espessura macular central foi semelhante nos dois grupos na última consulta (407,27 ± 99,08 µm e 376,66 ± 74,71 µm, p=0,66). A melhor acuidade visual corrigida aumentou significativamente em ambos os grupos. Não houve diferença entre os dois grupos quanto à melhor acuidade visual corrigida inicial e final.

**Conclusão:**

O tratamento com aflibercepte intravítreo foi altamente eficaz em melhorar a acuidade visual corrigida e a espessura macular central no edema macular induzido pela oclusão de um ramo da veia retiniana, independentemente da presença ou não de um descolamento seroso da retina.

## INTRODUCTION

Retinal vein occlusion (RVO) is the most common type of retinal vascular disorder after diabetic retinal disease and one of the most common causes of sudden painless unilateral vision loss^(1)^. The two types of RVO are the defined central RVO (CRVO) and retinal vein branch occlusion (BRVO). BRVO accounts for approximately 80% of the estimated 16 million cases of RVO worldwide^(2)^. Macular edema (ME), which occurred in approximately 60% of the total cases, is the most common cause of vision loss in patients with RVO^(3)^. It may occur secondary to fluid leakage from vessels in response to the increased intravascular hydrostatic pressure due to the occlusion.

In patients with BRVO, retinal ischemia causes the secretion of inflammatory mediators such as the endothelial growth factor (VEGF) and interleukin 6^(4)^. Increased cytokine level has been identified as the chief cause of the disruption of the blood-retina barrier, endothelial dysfunction, and increased vascular permeability^(4,5)^. In addition, the levels of soluble vascular endothelial growth factor receptors 1 and 2, other growth factors (e.g., placental and platelet-derived growth factors), intercellular adhesion molecules, monocyte chemoattractant protein, and various interleukins (e.g., IL-8, IL-12, and IL-13) detected in the aqueous humor have been significantly higher in RVO and correlated with ME^(5)^.

Although the standard treatment for ME in cases of BRVO was previously grid laser photocoagulation^(6)^, advances in the pharmaceutical industry and retinal imaging technologies coupled with better understanding of the pathogenesis of ME have altered the treatment protocol. Owing to the detection of increased levels of VEGF and other inflammatory factors in aqueous humor, anti-VEGF agents and steroids have replaced grid laser photocoagulation in the treatment of ME to ensure the anatomical resolution, stabilization, and improvement of visual acuity (VA). Various methods involving the use of intravitreal dexamethasone implants and anti-VEGF agents such as ranibizumab, bevacizumab, and aflibercept are currently used to treat ME in BRVO^(6)^, and those involving the use of intravitreal anti-VEGF agents have been especially preferred for treatment.

Optical coherence tomography (OCT), an indispensable technology in day-to-day ophthalmological examinations in the past decade, has allowed ME to be better defined and is useful for guiding the treatment and follow-up of patients with ME^(3,7)^. Studies have shown that ME in BRVO is associated with cystoid ME (CME), serous retinal detachment (SRD), and inner retinal thickening and found in 15-80% of patients with SRD BRVO associated with inflammatory, neoplastic, and ischemic diseases^(3,7,8)^. Although the mechanism behind SRD remains incompletely understood, it is known to be associated with vascular leakage exceeding the drainage capacity of the retina and, in turn, to cause fluid accumulation in the subretinal distance^(9)^. Some studies have shown that SRD also damages the retinal pigment epithelium (RPE) and neurosensory retina, thereby leading to poor prognoses for BRVO^(10,11,12)^.

As the success of treating ME due to BRVO depends on the disappearance of the edema and increased vision, the aim of our study was to evaluate the effect of intravitreal aflibercept injection on best-corrected VA (BCVA) and central macular thickness (CMT) in the treatment of ME in eyes with and eyes without SRD secondary to BRVO.

## METHODS

The sample included in this retrospective study included 37 patients (37 eyes) diagnosed as having ME secondary to BRVO in the Department of Ophthalmology at Kutahya University of Health Sciences between January 2015 and January 2019. The study, after approval by the local ethics committee, was conducted in accordance with the Declaration of Helsinki.

The records of 52 patients with BRVO were retrospectively reviewed, and 37 patients who met the inclusion criteria formed the study sample, were monitored regularly for 12 months from the initial diagnosis, and provided complete data for analysis. All the patients were naive to treatment, and only one eye of each participant was studied.

The major inclusion criterion for our study was the presence of SRD and/or CME on OCT, with ME involving the center of the fovea with a minimum CMT of 300 µm. The other inclusion criteria were VA <0.8 according to the Snellen chart, age >30 years, mean macular thickness of at least 300 µm in two sections as measured on OCT images, sufficient pupil dilatation, and willingness to participate. By contrast, the exclusion criteria were ME due to any cause other than BRVO, age-related macular disease, previous ocular surgery (including patients with cataract surgery in the past 6 months), neovascularization, diabetic retinopathy, history of laser photocoagulation or intravitreal injection, current use of systemic steroids, clinically significant media opacity, vitreomacular traction on OCT, history of ocular inflammation, and pronounced retinal bleeding, including macular and foveal bleedings.

In all the examinations, BCVA was measured with a Snellen chart, and the logarithm of the minimum resolution angle (logMAR) was converted into units for statistical analysis. The anterior and posterior segments were examined with a biomicroscope, and intraocular pressure was measured with a non-contact tonometer. Fluorescein angiography (FA) was performed to detect ischemia and neovascularization, and each case of BRVO with a nonperfused area of <5 disk diameters as measured using FA was defined as non-ischemic BRVO. Eyes with CMTs of at least 300 µm on OCT (Heidelberg Engineering, Heidelberg, Germany; 3D-OCT 1000, Topcon, Tokyo, Japan) were included in the sample, at which point treatment approaches were determined.

Representing the average retinal thickness of the central 1 mm of the macula and automatically measured on OCT, CMT was defined as the vertical distance between the internal limiting membrane and the RPE in the central fovea. By contrast, CME referred to the hyporeflective intraretinal cavities radiating from the center of the macula in cross-sectional scans. SRD referred to the presence of nonreflective cavities with minimal shadowing of the underlying tissues due to subretinal fluid accumulation and the consequent detachment of the neurosensory retina. On the basis of these definitions, the patients were divided into two groups according to the patients’ spectral domain OCT findings regarding SRD^(13)^ and CME as follows: patients with CME only (non-SRD Group, n:22) and patients with SRD (SRD Group, n:15). All the eyes in the SRD Group also had CME.

After the patients were informed about the intravitreal injection process and possible complications and signed the informed consent form, injections were administered in the operating room. After topical anesthesia, at least 3 minutes of asepsis and antisepsis were applied with 10% povidone iodine to the eye area and 5% povidone iodine in the injection site. Next, 2 mg/0.05 mL aflibercept (Eyle, Regeneron Pharmaceuticals, Inc., Tarrytown, NY, USA, and Bayer Pharma AG, Berlin, Germany) was intravitreally injected with a 30-gage needle in the pars plana. For 1 week after each injection, topical 0.5% moxifloxacin (Vigamox) was prescribed to be applied 4 times daily.

After one intravitreal dose of aflibercept, a pro re nata regimen was followed. The criteria for the reinjection of aflibercept included morphologically evident intraretinal edema or subretinal fluid observed on OCT, persistent edema with a CMT of at least 300 mm, recurrent ME with a CMT increased by at least 50 mm to values obtained from the previous examination period, or a subjective decrease in VA from the previous follow-up visit. After the initial injection, the control treatments were administered at months 1, 2, 3, 6, and 12, and a detailed ophthalmologic examination was repeated for all the controls. The principal outcome measures were the changes in BCVA and CMT from baseline to the final visit.

SPSS version 15.0 was used for the statistical analysis, an independent-samples *t* test was used to compare the two groups after analyzing the normal distribution of the data, and a paired-sample *t* test was performed for post-treatment comparison.

## RESULTS

For the 37 eyes of 37 patients who met the inclusion criteria and were enrolled in the study, the minimum follow-up period was 12 months, and the BRVO duration in all the patients was <2 months. Occurring in 60% of cases, hypertension was the most common systemic disease. At baseline, 15 patients (40.5%) had SRD (i.e., SRD Group), while 22 (59.5%) had CME without SRD (i.e., non-SRD Group). The groups did not significantly differ in terms of age, sex, laterality, diagnosis with hypertension, or BRVO duration (Table 1).

**Table 1:** Clinical characteristics of the patients at baseline

	SRD	Non-SRD	p value
Sex, n (female/male)	15 (8/7)	22(12/10)	0,32
Age (y), mean ± SD	65,93 ± 7,99	62,81 ± 9,78	0,31
Mean VA (logMAR)	0,97 ± 0,41	0,77 ± 0,47	0,24
Right eye/left eye	6/9	9/13	0,26
Mean CMT (µm,mean ± SD)	811,73 ± 220,68	667,90 ± 220,68	0,042
Duration of BRVO(weeks) mean ± SD	5,06 ± 2,0	4,50 ± 1,97	0,40

BRVO= branch retinal vein occlusion; CMT= central macular thickness; F= female; logMar= logarithm of the minimum angle of resolution; M= male; SRD= serous retinal detachment; VA= visual acuity.

Although BCVA was initially worse in the SRD Group (0.97 ± 0.41) than in the non-SRD Group (0.77 ± 0.47), the difference was not statistically significantly different (p>0.05). CMT, however, was significantly higher (p<0.05) in the SRD Group (811.73 ± 220.68) than in the non-SRD Group (667.90 ± 220.68). After the first aflibercept injection, the mean BCVA in the SRD Group increased from 0.97 ± 0.41 logMAR to 0.75 ± 0.47 logMAR (p<0.05), whereas the CMT decreased from 811.73 ± 220.68 µm to 531.93 ± 203.27 µm (p<0.001). In the non-SRD Group, the mean BCVA increased from 0.77 ± 0.47 logMAR to 0.49 ± 0.50 logMAR (p<0.05), whereas the CMT decreased from 667.90 ± 220.68 µm to 396.36 ± 88.00 µm (p<0.001).

**Figure 1:**
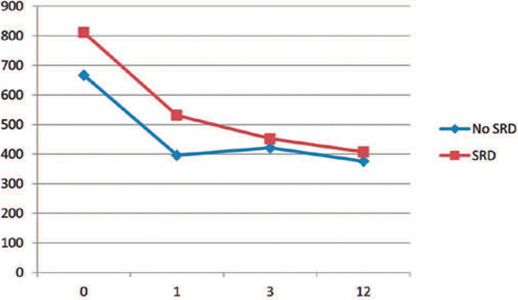
Changes in central macular thickness (CMT) from baseline CMT showing a statistically significant decrease in both groups (p<0.001). However, no significant diference was found between the two groups in the third month control.

**Figure 2:**
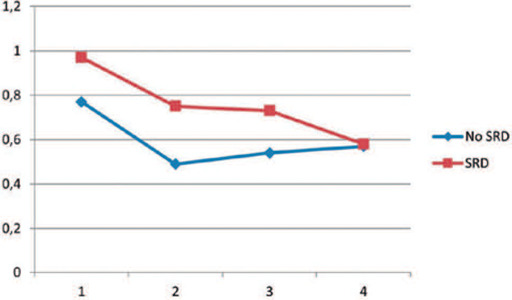
Changes in best-corrected visual acuity (BCVA) from baseline, measured using the logarithm of the minimum resolution angle chart, showing a statistically significant improvement in both groups (p<0.001). However, no signficant diference was found between the groups.

Although the difference in CMT continued into the control at month 1, no significant difference emerged between the groups in the control at month 3 (Figure 1, Table 2). In the final control, the BCVAs were 0.58 ± 0.48 and 0.57 ± 0.45, and the CMTs were 407.27 ± 99.08 and 376.66 ± 74.71 in the SRD and non-SRD groups, respectively. No significant difference in final BCVA (p=0.99) and CMT were found between the groups (p=0.66, Figures 1 and 2).

**Table 2:** Intergroup and intragroup comparisons of central foveal thickness and best-corrected visual acuity measurement

	Baseline	Month 1	Month 3	Month 12	p
CMT (µm)					
No-SRD	667,90 ± 220,68	396,36 ± 88,52	421,36 ± 121,01	376,66 ± 74,71	<0,001
SRD	811,73 ± 220,68	531,93 ± 132,14	452,57 ± 127,78	407,27 ± 99,08	<0,001
P values	0,04	0,02	0,55	0,66	
BCVA (logMAR)					
No-SRD	0,77 ± 0,47	0,49 ± 0,50	0,54 ± 0,44	0,57 ± 0,45	<0,001
SRD	0,97 ± 0,41	0,75 ± 0,47	0,73 ± 0,52	0,58 ± 0,48	<0,001
P values	0,24	0,15	0,26	0,99	

CMT= central macula thickness; BCVA= best-corrected visual acuity; SRD= serous retinal detachment.

Peripheral retinal nonperfused areas, all <5 disk diameters away from the macula, were found in two eyes in the SRD Group and in five eyes with FA in the non-SRD Group. Argon laser scatter photocoagulation was performed in those areas 1 week after the first injection in accordance with the WAVE study protocol^(14)^, and the effects were compared against the developments in the patients who did not receive laser therapy. Ultimately, no significant difference in BCVA or CMT was found (p>0.05). Simple and multiple linear regression analyses using the enter method to detect the effective factor for the final BCVA revealed that the change in baseline BCVA was the most valuable predictive factor of VA (β =0.800, p<0.001 and β =0.921, p<0.05, respectively) (Table 3).

**Table 3:** Predictive factors of visual gain

	Simple regression analysis		Multiple regression analysis	
Predictive factor	Beta coefficient	p	Beta coefficient	p
SRD	0,04	0,990	0,130	0,548
Initial CMT	0,131	0,640	-0,171	0,474
Status of LFK	-0,258	0,353	0,172	0,493
Initial BCVA	0,800	0,000	0,921	0,003

CMT= central macular thickness; BCVA= best-corrected visual acuity; LFK= laser photocoagulation; SRD= serous retinal detachment.

After the third injection, one patient developed a high intraocular pressure. No endophthalmitis or vitreous hemorrhaging was observed during the injections.

## DİSCUSSİON

The increased use of OCT and technological advancements have allowed clearer definitions of the anatomical appearance of ME^(12,13)^, the most common cause of visual impairment in cases of BRVO^(6)^. After the onset of BRVO, sponge-like retinal swelling, CME, and development of SRD have also been reported as structural changes in patients with ME^(3,8,12,13)^. SRD, reported in 15.0%-80.8% of patients with BRVO^(12)^, was found in 40.5% of the patients in our study.

SRD, which we initially detected with OCT, has been identified as a poor prognostic factor of RVO^(10,11,12)^. In SRD, fluid accumulates between the neurosensory retina and the RPE, in areas that thus appear as hyporeflective spaces on OCT. The currently accepted theoretical pathogenesis of the disorder suggests that the leakage from the capillaries affected by RVO into the subretinal space exceeds the capacity for drainage^(12)^. The pathogenesis of SRD has also been associated with hemodynamic overload and impairment of RPE function^(13)^. Although it is known as an RPE-resistant tissue, proinflammatory mediators such as VEGF may disrupt its structure and function in response to ischemia^(15)^, and VEGF-mediated permeability may consequently cause RPE barrier dysfunction^(13,15)^. In turn, the excessive increase in vascular permeability secondary to VEGF upregulation may contribute to the development of SRD in patients with BRVO^(16)^.

Some studies have shown that inflammatory factors are associated with the pathogenesis of SRD^(16,17,18)^. Dacheva et al. found that inflammatory cytokines correlated with CMT and the extent of SRD^(17)^, while Park et al. observed that VEGF levels in the aqueous humor were higher in patients with BRVO involving SRD than in those with BRVO without SRD^(18)^. Noma et al. found that the vitreous fluid levels of VEGF and soluble intercellular adhesion molecule 1 were higher in patients with BRVO involving SRD than in those with BRVO without SRD^(16)^. In their study, they examined concentrations of intraocular cytokines before and after an intravitreal injection with bevacizumab in patients with BRVO and compared the results in terms of treatment efficacy. Some angiogenic (e.g., VEGF) and proinflammatory factors were significantly suppressed after the injection. The clinical efficacy of the treatments, measured as change in VA with OCT, aligned with the degree of suppression of the increases in cytokine levels^(5)^. In our study, these relationships can explain the initially high CMT in the SRD Group and the lack of a statistically significant difference between the groups by providing more anatomical improvement after anti-VEGF treatment.

Noma et al. also reported significantly worse BCVA in the group of patients with SRD than in the group without SRD and that CMT was also higher in the former group^(16)^. In view of those findings, they hypothesized that the worse BCVA in the cases of SRD may have been associated with the photoreceptor cell damage caused by the macular detachment and functional impairment secondary to ischemia amid the high levels of VEGF in vitreous fluid^(16)^. They also detected no significant difference between the groups in terms of macular sensitivity, whereas the initial vision was worse and the CMT was higher in the group of patients with SRD^(11)^. The microperimetric results additionally showed that SRD does not affect macular sensitivity nor does not necessarily lead to poor visual prognoses^(11)^. These results suggest that SRD may relate to a decrease in VA together with CME, as nearly all the patients with SRD had CME as well^(11)^. Initial visual impairment may be due to the mechanical photoreceptor cell damage caused by the retinal detachment and can be reversed once SRD is resolved^(16)^. Previous results support the finding that after SRD resolution, patients with SRD had a better visual prognosis than previously determined^(16)^. In our study, the absence of functional and anatomical differences in both groups after treatment suggests that visual potential, independent of SRD, can be preserved in those patients, as they return to the normal retinal structure after treatment with aflibercept.

Treatment for ME secondary to BRVO has greatly improved recently with the introduction of a therapy based on anti-VEGF molecules and steroid injection^(19)^. VEGF inhibitors have particularly revolutionized the treatment of BRVO associated ME, a VEGF-sensitive condition. Moreover, evidence shows that VEGF is an important tool for treating ME in BRVO^(4,20)^ and that the resolution of ME and visual development can occur in response to the pharmacological inhibition of VEGF^(4)^. Currently, the most commonly used anti-VEGF drugs are bevacizumab (Avastin), aflibercept (Eylea), and ranibizumab (Lucentis)^(5,6,21,22)^. The known members of the VEGF family for ocular diseases are VEGF-A, VEGF-B, and placental growth factor, and aflibercept is a recombinant fusion protein that binds placental growth factor and all isoforms of VEGF-A and-B for the longest half-life in circulation among aflibercept-based anti-VEGF agents^(23)^. Shown to have a higher affinity for VEGF-A than bevacizumab and ranibizumab in vitro^(24)^, aflibercept was approved for this indication after the release of the results of COPERNICUS and GALLİLEO, the two most important randomized controlled clinical studies that evaluated its use in cases of ME secondary to RVO^(25,26)^.

Given the evidence that suggests the relationship between the development of SRD and VEGF^(16,17,18)^, studies have been conducted to investigate the effectiveness of anti-VEGF treatments in patients with BRVO who develop SRD^(27,28,29,30)^. Dogan et al. evaluated the effect of a single injected dose of ranibizumab on BCVA and CMT in the treatment of CME in eyes with and eyes without SRD secondary to BRVO. In their study, mean reductions of 72 and 46 µm were observed in the SRD and non-SRD groups, respectively, at the end of the first month^(27)^. Ultimately, they concluded that BCVA and CMT could be treated with an intravitreal injection of ranibizumab in patients with BRVO with and those with BRVO without SRD, although the anatomical improvement was greater in the SRD group. Similarly, in our study, the decrease in CMT was higher in the SRD Group at the end of month 12 (Table 2).

In their study on the effectiveness of bevacizumab in patients with ME involving SRD secondary to BRVO, Poon et al. observed that after 6 months, BCVA increased by 55 letters and CMT decreased by 412 µm. Beyond that, the patients with SRD showed greater functional and morphological improvements at 6 months after primary therapy with bevacizumab^(28)^. At the last follow-up, no statistically significant difference emerged between the groups in terms of BCVA, CMT, or the number of injections^(28)^. As in the study by Poon et al., in our study, we found no significant difference between the two groups in terms of initial BCVA, and CMT was higher in the SRD Group, although the difference disappeared after 3 months of treatment.

Gallego-Pinazo et al. compared the response to ranibizumab between patients with BRVO involving SRD and those with BRVO without SRD and found that both BCVA and CMT improved significantly in the patients with SRD after an average of 5.0 ranibizumab injections over 12.5 months. By contrast, the patients without SRD received an average of 4.3 injections^(29)^. Gallego-Pinazo et al. analyzed the effect of SRD on the visual prognoses after repeated intravitreal treatment with ranibizumab and reported that SRD may be a basic predictive factor of ranibizumab treatment outcomes in patients with BRVO regardless of the number of treatments^(29)^. Similarly, in our study, the presence of SRD did not affect the number of injections.

Küçük et al. included only cases with SRD in a study of patients with BRVO and compared two anti-VEGF agents. Finding greater improvements in CMT and BCVA with aflibercept than with ranibizumab in the first 3 months of treatment, they proposed that aflibercept is more effective than ranibizumab in the initial treatment of ME with SRD due to the pharmacokinetic properties of aflibercept molecules. The differences disappeared over the next 9 months, and changes in CMT and BCVA were similar across the groups at all other visits. The mean BCVA increased by 14.06 ± 4.45 letters with ranibizumab and by 15.10 ± 5.18 letters with aflibercept, whereas CMT decreased by 294.21 ± 73.85 µm with ranibizumab and by 304.03 ± 76.66 µm with aflibercept, all by the end of the 12-month period^(30)^. In the first 6 months of treatment, the patients with or without SRD received an average of 3.90 and 3.70 injections, respectively, but no statistically significant differences emerged^(30)^.

In our study, the difference in CMT between the SRD Group (396.36 ± 88.52 µm) and non-SRD Group (531.93 ± 132.14 µm) at the first examination after 12 months of follow-up continued into control period at month 1 but disappeared at month 3, up to 421.36 ± 121.01 µm in the SRD Group and 452.57 ± 127.78 µm in the non-SRD Group. Although BCVA improved with treatment in both groups, no significant difference appeared between the groups during the 12-month period. The number of injections were 3.4 ± 1.5 in the SRD Group and 3.0 ± 1.5 in the non-SRD Group, which are not statistically different.

Anti-VEGF therapy has been shown to provide anatomical and functional improvements in patients with SRD secondary to BRVO. As shown in such studies, a strong association between VEGF intensity and SRD^(5,16,17,18)^ suggests that response to treatment can be increased and anatomical success can be pronounced^(27,28,29,30)^. Our study supports these results.

The major limitations of our study include its retrospective design, small samples in the groups, and the lack of functional mapping by microperimetry (i.e., macular sensitivity). Randomized, controlled, and comparative trials, with higher numbers of eyes and longer follow-up periods are thus needed to confirm the role of SRD as a predictive factor of BRVO with ME treated with aflibercept.

In conclusion, although no significant difference emerged in the groups between the first and final BCVA, the initial CMT was significantly higher in the SRD Group, although the difference disappeared at month 3 after treatment. At the end of month 12, we clearly observed that treatment with aflibercept was highly effective in improving BCVA and CMT in the BRVO-induced ME independent of SRD. The presence of SRD in BRVO-induced ME treated with aflibercept did not affect the anatomical and visual outcome or increased the number of injections. However, the patients with SRD demonstrated a more significant improvement in macular morphology than those without SRD. In addition, the baseline CMT was quite high in both groups. Considering these real-life data, we believe that aflibercept was visually and anatomically successful despite the low number of injections in both groups.
